# IgG4-related sclerosing cholangitis mimicking cholangiocarcinoma

**DOI:** 10.1093/jscr/rjad621

**Published:** 2023-11-22

**Authors:** Wei R Ng, Ngee-Soon Lau, Mitali Fadia, Sivakumar Gananadha

**Affiliations:** Department of General Surgery, North Canberra Hospital, Bruce, ACT 2617, Australia; Hepatopancreatobiliary Unit, Canberra Hospital, Garran, ACT 2605, Australia; Department of Anatomical Pathology, Canberra Hospital, Garran, ACT 2605, Australia; Department of General Surgery, North Canberra Hospital, Bruce, ACT 2617, Australia

**Keywords:** IgG4-related sclerosing cholangitis, cholangiocarcinoma, autoimmune disease

## Abstract

A man in his 70s presented to the emergency department with painless obstructive jaundice. Initial blood test results show a predominantly cholestatic picture with elevated tumour markers, and imaging findings are concerning for a pancreatic head neoplasm or cholangiocarcinoma with involvement of the entire common bile duct. The patient underwent staging laparoscopy and biopsies including peritoneal washing, but did not identify any features of malignancy. Immunoglobulin G and immunoglobulin G4 testing were subsequently tested and shown to be elevated. The provisional diagnosis of immunoglobulin G4-related sclerosing cholangitis was made, and steroid treatment was empirically started. Treatment with steroids was successful, with complete resolution of symptoms and abnormal imaging findings and near complete resolution of liver function test results after 1 month.

## Introduction

This case report aims to highlight the classical presentation of immunoglobulin G4 (IgG4)-related sclerosing cholangitis mimicking as cholangiocarcinoma. We discussed the latest literature regarding the disease, the diagnostic criteria, the treatment options, and the common pitfalls of an incorrect diagnosis.

## Case report

**Figure 1:**
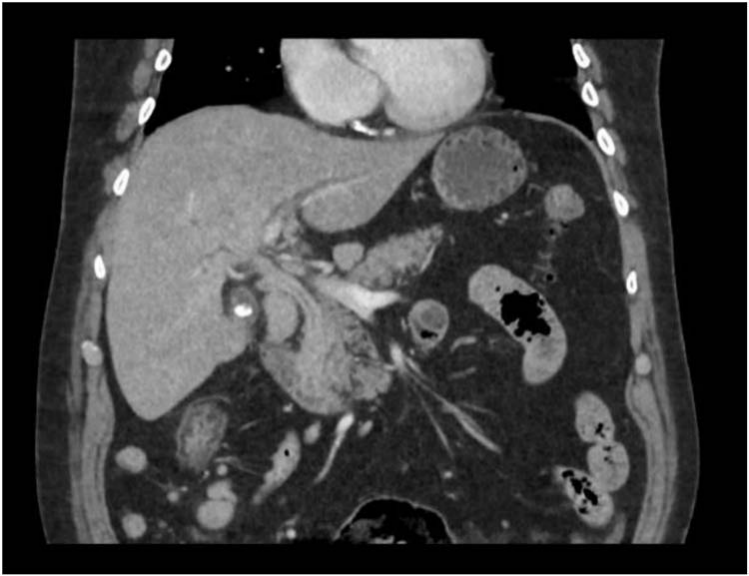
CT scan showing thickening and enhancement of the entire extrahepatic common bile duct with intrahepatic duct dilatation.

**Figure 2:**
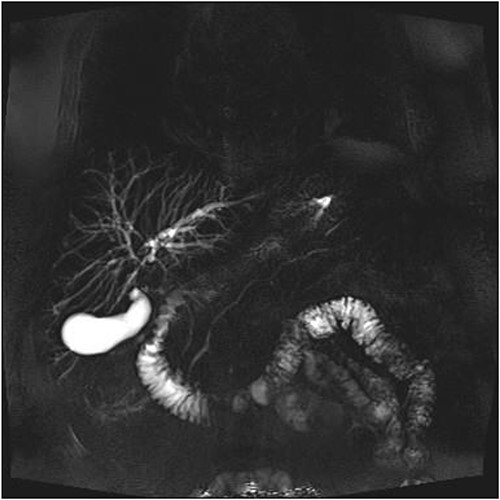
MRCP showing involvement of entire common bile duct.

**Figure 3:**
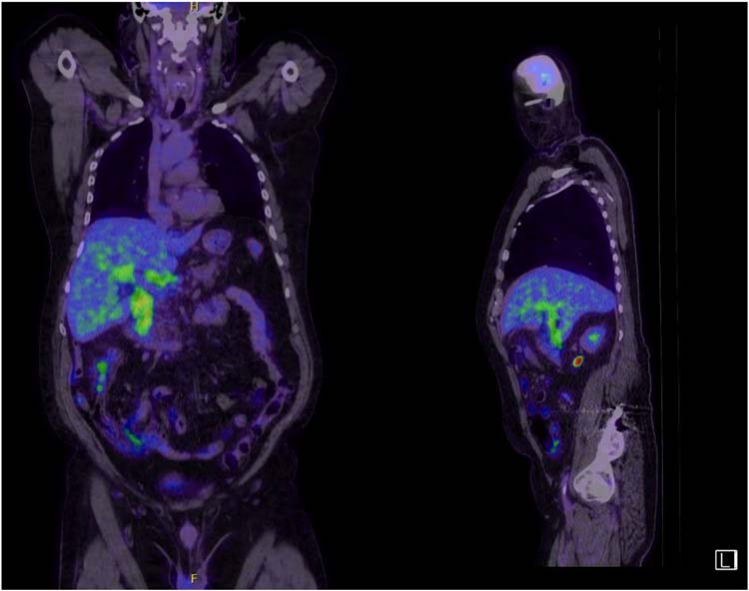
Abnormal FDG uptake on PET scan extending from intrahepatic duct to distal common bile duct with uptake in the tail of pancreas.

A man in his 70s presented to the emergency department with painless obstructive jaundice with dark urine and pale stools. The patient denied having any fever, nausea, vomiting, or weight loss. Liver function test progressively worsened during the admission peaking at a bilirubin of 287 umol/L (normal 2–20), alkaline phosphatase 694 U/L (normal 30–110), and alanine aminotransferase 160 U/L (normal < 40). Initial assessment on computed tomography and ultrasound raised concerns for primary pancreatic head neoplasm or cholangiocarcinoma (Fig. 1). He subsequently underwent magnetic resonance cholangiopancreatography (MRCP), which showed involvement of the entire common bile duct with no definite stricture or dilatation and no discrete pancreatic head mass (Fig. 2). Positron emission tomography showed abnormal uptake in the intrahepatic and extrahepatic bile ducts, focal uptake in the tail of the pancreas, and lymph nodes in the porta hepatis, retroperitoneum, and right iliac fossa (Fig. 3). Tumour markers revealed a carbohydrate antigen 19-9 (CA19-9) of 3286 kU/L (normal < 34).

The patient underwent a staging laparoscopy, which found a distended gallbladder with bulky portal lymph nodes but no evidence of liver, peritoneal, or omental metastasis. Liver biopsy showed portal tract-based granulomatous cholangitis and IgG4 immunohistochemistry testing was negative on the liver biopsy. Portal lymph node biopsy was benign, and no malignant cells were identified in peritoneal washing. Serum immunoglobulin G (IgG) testing revealed an elevated total of 30.53 g/L (normal 6.5–15.2) and IgG4 of 3.03 g/L (normal 0.04–0.86). He remained well with no evidence of cholangitis despite no antibiotics being administered.

A presumptive diagnosis of IgG4-related sclerosing cholangitis was made, and he was started empirically on 40 mg of oral Prednisolone for 4 weeks.

**Figure 4:**
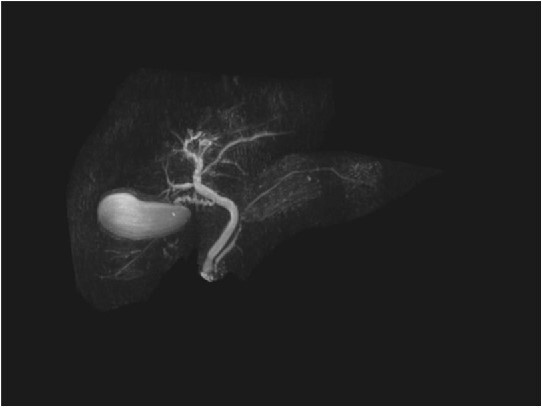
Follow-up MRCP with resolution of common bile duct pathology.

Repeat liver function test 1 month later showed dramatic improvement, with bilirubin level at 25 umol/L (normal 2–20), total IgG 13.23 g/L (normal 6.5–15.2), IgG4 1.45 g/L (normal 0.04–0.86), and CA19-9 44 (normal < 34). The steroids were gradually tapered and ceased. Repeat MRI 4 months later showed a normal biliary system with complete resolution of the common bile duct pathology (Fig. 4).

## Discussion

IgG4-related sclerosing cholangitis is a subtype of a multiorgan IgG4-related sclerosing disease, which responds well to steroid therapy. It is characterized by the presence of a biliary stricture, frequently associated with other fibrosing diseases such as autoimmune pancreatitis, and by the presence of IgG4-positive plasma cells and T-lymphocyte in various organs [1, 2]. IgG4-related sclerosing disease is more commonly seen in older men, and can affect multiple organs including the pancreas, bile duct, gallbladder, salivary gland, retroperitoneum, kidney, lungs, and prostate [2]. The autoimmune disease appears to be linked to blue collar workers with chronic occupational exposure to antigens, as well as links to patients with a history of atopy with peripheral eosinophilia and elevated immunoglobulin E [3]. Long-term management planning is important, as one study quoted a relapse in 12 out of 17 patients after stopping steroids, and a further two patients relapsed whilst being on a steroid wean. The current consensus for treatment dosage and duration is 0.6 mg/kg/day for 2– 4 weeks, with a gradual taper of 2.5–5 mg/day over 2–3 months [4]. An alternative to steroids used in cases of recurrent relapses that has been described are immunomodulators such as azathioprine, mycophenolate, and rituximab, as well as decompressive biliary stenting in the setting of symptomatic obstructed biliary strictures [5, 6].

Diagnosis is challenging as primary sclerosing cholangitis, IgG4-related sclerosing cholangitis and cholangiocarcinoma can mimic each other with each requiring a different treatment plan. An intraductal ultrasonography and transpapillary biopsy may be useful in diagnosing IgG4-related sclerosing cholangitis with IgG4 immunostaining [7]. Cytological samples from brush cytology and an analysis of the biliary fluid are also useful tools to exclude malignancy [3]. It is diagnostic to have more than 10 IgG4-positive cells per high power field in a biopsy or more than 50 IgG4-positive cells per high power field in a resection specimen. In our case report, however, IgG4 immunohistochemistry is almost negative. According to Culver *et al*., this could be because of a patchy distribution of the disease process, insufficient tissue sampling from the biopsy, and fibrotic phenotype [3]. A diagnostic criterion was recently published by Nakazawa *et al*. [8] based on the evidence of narrowing of the bile duct and thickening of the bile duct wall on imaging, serological, and pathological findings of elevated IgG4 or lymphoplasmacytic infiltration and fibrosis, evidence of other organ involvement, and effectiveness of steroid therapy.

It is also worth noting, however, that there is a possibility of carcinomatous change in a patient with autoimmune disease, as reported by Oh *et al*. Their patient was a 59-year-old man with features of autoimmune pancreatitis, but histology showed carcinomatous change with abundant IgG4-positive cells in the common bile duct [9]. This was likely because of the persistent biliary inflammation from mast cell activation leading to metaplasia and subsequent dysplasia [10]. It is also possible to misdiagnose metastatic cholangiocarcinoma as autoimmune cholangitis in the setting of slightly elevated serum IgG4 and CA19.9 as reported by Zhang *et al*. In their study, they have highlighted the importance of follow-up tumour marker tests and repeat imaging after immunosuppressive therapy was started [11]. The possibility of malignancy should therefore not be completely discounted in patients with suspected autoimmune cholangitis.

Kubota *et al*. reported in their study that 139 out of 924 patients with IgG4-related sclerosing cholangitis subsequently developed malignancy, with relapses being an independent risk factor, highlighting the importance of surveillance. The study acknowledges that whilst there is ongoing debate in regards to the ideal duration of therapy to prevent relapses, a maintenance steroid treatment period of 3–5 years has been suggested to yield a longer relapse-free period [12].
